# The Ineffable Pills

**Published:** 1861-01

**Authors:** Gulliver Sancho Panza


**THE INEFFABLE PILLS.**—I, John Lubberlee, was supposed to be in the last stage of consumption in the year eighteen hundred and forty-eight, suffering at the same time under a severe attack of rheumatism, liver complaint, plumbago, gravel, dropsy and cholera-morbus. Simultaneously, also, I took the yellow-fever and small pox. The latter assuming the chronic form of scrofula, completely destroyed my lungs, liver, spinal marrow, nervous system, and the entire contents of my cranium. I got so low, that I did not know my brother-in-law when he came to borrow some money. For three months I swallowed nothing but twenty boxes of the "Ineffable Pills," which effected an immedate cure in two weeks. Sworn and subscribed to, etc.

P. S. N. B—My late Uncle Bacchus Pottinger, was afflicted so long with the gout, contracted by living too much on bears' meat and alligators' eggs, that life became a burden to him. He took only four boxes of the "Ineffables," and life was a burden to him no longer. GULLIVER SANCHO PANZA.

But not to deal too much in pills we will make an extract from the Brief-Book of a Lawyer about

